# Effects of Defoliation Timing and Intensity on Yield Components and Grain Quality of Quinoa (*Chenopodium quinoa* Willd.)

**DOI:** 10.3390/plants14030413

**Published:** 2025-01-30

**Authors:** Maria I. Ahumada, Nathaniel B. McCartney, Rodrigo A. Chorbadjian

**Affiliations:** 1 Departamento de Ciencias Vegetales, Facultad de Agronomía y Sistemas Naturales, Pontificia Universidad Católica de Chile, Santiago 7820436, Chile; miahumad@uc.cl; 2 Department of Entomology, The Pennsylvania State University, University Park, PA 16802, USA; nbm2@psu.edu

**Keywords:** tolerance, compensatory growth, overcompensation, grain quality, herbivory, saponins

## Abstract

Understanding plant tolerance to defoliation is crucial for sustainable pest management and reducing pesticide use in food production. This study explores quinoa’s (*Chenopodium quinoa* Willd.) responses to foliar damage, which have been largely unexamined. Over two seasons, quinoa plants were subjected to mechanical defoliation at different pre-reproductive stages and intensities (0–60%) in the first season, and both mechanical and insect-induced (*Trichoplusia ni* (Hübner), Lepidoptera: Noctuidae) defoliation in the second. The results showed that quinoa plants consistently tolerated defoliation without reductions in grain number, weight, above-ground biomass, or harvest index. These compensatory responses were independent of the defoliation method, timing, or intensity. In the first season, overcompensatory effects were observed, leading to increased plant biomass at 60% early defoliation and 40% late defoliation. Additionally, early defoliation at 20% and 60%, as well as late defoliation at 60%, led to an increase in grain number without affecting grain weight. Defoliation did not significantly alter the phenolic content, sapogenins, or antioxidant capacity of the grains, preserving their phytochemical quality. These findings enhance the understanding of quinoa’s resilience to herbivory, suggesting that it can withstand defoliation stress without compromising yield or quality.

## 1. Introduction

Quinoa (*Chenopodium quinoa* Willd.) is a crop of growing interest due to the nutritional quality of its grains, as well as its tolerance to water and saline stress [1,2]. Although it is mainly grown in the highland ecoregion of South America, its cultivation has been spreading to other regions of the world, including Asia, Africa, Europe, and North America, where it holds the promise of providing a local production option in environments that are marginal for more traditional crops [3,4]. This increase in interest in the crop has also sparked growing interest in carrying out research and development to improve production, although there are still important knowledge gaps regarding pest management issues [5,6].

A wide variety of insects can feed on quinoa, with Lepidoptera larvae being the ones most frequently reported to defoliate and cause damage to grain production [5,7,8,9]. Defoliations of more than 50% have been reported [7], and depending on the density of insect larvae, they can cause significant yield loss [8]. This causes concern among farmers who would benefit from greater knowledge in managing these pests in an integrated and sustainable manner.

Damage caused by defoliation can be expected to negatively affect yield. However, a damage-susceptible type response is not what always occurs since plants have the capacity to produce growth responses that compensate for the biomass lost due to defoliation [10]. Thus, plants can express growth responses that compensate for damage, a response called tolerance, or they can even overcompensate, in which greater growth is observed in damaged plants compared to undamaged plants [10,11,12,13,14,15]. Studying this type of response is of both ecological interest due to its potential effect on plant fitness, as well as agronomic interest due to its potential effect on yield components and, particularly, grain production. Damage-tolerant plants could aid integrated pest management by reducing the need for insecticide use [16,17,18].

Damage tolerance responses may depend on mechanisms related to the acquisition and redistribution of resources, such as increased photosynthetic rate, the utilization of stored reserves, changes in plant phenology, and architectural traits that allow for regrowth [19]. In a scenario of limited resources, the efficiency of the plant for the redistribution of resources is relevant since more efficient plants would allow for productivity to increase. In this context, the harvest index reflects the proportion of biomass destined for grains with respect to the total, being an indicator of crop efficiency [20]. In quinoa, the harvest index averages 0.33 [21,22], although there is variation, and in specific cases, it can even reach 0.57 [23]. As the production environment has important effects on this variability [22,23], it is interesting to identify and characterize possible sources of variation, of which the possible effect of defoliation has not been studied.

The timing of defoliation in relation to the phenological stage of the plant may be relevant to the plant’s ability to recover from damage [18,24,25]. If damage occurs in the early stages, the plant is expected to have time to recover and compensate for the loss, but if damage occurs during the flowering and seed production stage, it is more likely that the plant will not be able to compensate. This has been associated with the plant organ that was affected [18,25], architectural traits related to the ability to produce tillers or lateral branches [26,27], priming triggered by early damage [28], or shifts in the allocation pattern modulated by resource availability [29]. Empirical evidence in the case of quinoa is not available; however, it has been reported that amaranth (*Amaranthus cruentus* L.), a plant similar to quinoa, can tolerate high levels of defoliation in the vegetative stage but not in the reproductive stages [30]. In the case of quinoa, the critical period for yield determination has been recently identified to be during its reproductive phase [23]; thus, it is possible to expect fewer negative effects from stress during the vegetative phase.

The quality of quinoa grains is determined by physical attributes, including grain size and weight, as well as chemical characteristics, primarily the content of saponins, phenols, and antioxidant capacity. Quinoa is a valuable source of phenolic compounds and antioxidants, elements recognized for their benefits to human nutrition [31,32,33]. However, it also contains saponins, which are components with an unpleasantly bitter taste and which must be removed prior to human consumption. The saponin content in seeds typically ranges between 5 and 11 mg/g but can reach levels as high as 27 mg/g [34,35]. Chemically, saponins are polycyclic triterpene glycosides with up to seven central aglycone structures collectively referred to as sapogenins [36]. The primary sapogenins in quinoa include oleanolic acid, phytolaccagenic acid, hederagenin, and serjanic acid, while less abundant compounds include 3β-hydroxy-23-oxo-olean-12-en-28-oic acid, 3β-hydroxy-27-oxo-olean-12-en-28-oic acid, and 3β-23,30-trihydroxy-olean-12-en-28-oic acid [35,36].

Damage to the plant, whether mechanical or caused by insects, frequently activates or suppresses metabolic pathways, leading to the synthesis of secondary metabolites and altering primary metabolism and nutrient allocation patterns [37,38,39]. Mechanical defoliation is useful for standardizing methodologies; however, plant responses can be highly specific to the type of herbivore causing the damage, with potential differences or similarities to those elicited by mechanical defoliation [40,41]. How tolerance mechanisms and insect-induced effects influence the yield and quality of quinoa, as well as their contribution to agricultural productivity, remains largely unexplored.

The goal of this current study was to characterize quinoa growth compensatory responses to defoliation. The objective was to quantify yield components that included the number and weight of grains, above-ground biomass, and harvest index. To do this, plants in the first study season were subjected to mechanical defoliation at two times during pre-reproductive stages and at intensities of 0, 20, 40, and 60%. The effect of the defoliation method, either mechanical or with insects (*Trichoplusia ni* (Hübner), Lepidoptera: Noctuidae), was evaluated during a second study season that also included intensities of defoliation of 0, 20, 40, and 60%. Additionally, sapogenins, phenolic compounds, and the antioxidant capacity of the grains were analyzed in these second-season plants.

## 2. Results

**Figure 1:**
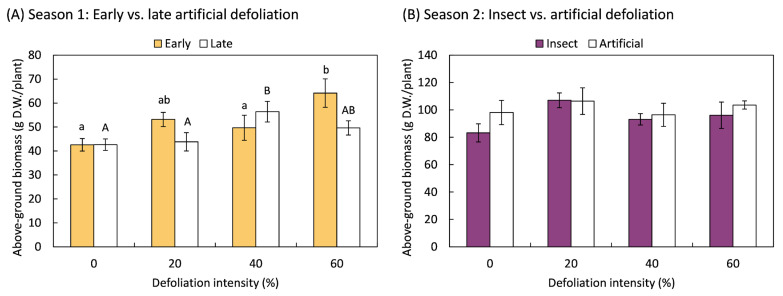
Total weight of the aerial part, including leaves, stems, and grains, of quinoa (*Chenopodium quinoa* Willd.) plants that were defoliated with intensities of 0, 20, 40, or 60% of leaf area removed during (**A**) early (8 BBCH-scale [42]) or late (50 BBCH-scale) developmental stages or (**B**) subjected to artificial or insect defoliation treatments applied at the 20 BBCH-scale stage. The bars represent the average of the treatments ± 1 SEM. Treatment means followed by the same letter are not statistically different (Fisher LSD, 0.05).

None of the defoliation treatments resulted in a decrease in above-ground biomass compared to non-defoliated plants, demonstrating the plants’ ability to compensate for leaf area loss due to defoliation in both Season 1 and Season 2 (Figure 1). Additionally, during Season 1, plants that were subjected to early defoliation treatments with an intensity of 60% (*F*_3,20_ = 4.17; *p* = 0.019) and late defoliation treatments with 40% (*F*_3,20_ = 3.32; *p* = 0.041) leaf area removal overcompensated for the damage, as the total above-ground biomass was greater than in the non-defoliated control (Figure 1A). In Season 2, the total biomass did not vary with respect to the method of defoliation, whether mechanical or by insects (*F*_1,70_ = 1.44; *p* = 0.235), the intensity of defoliation (*F*_3,70_ = 1.74; *p* = 0.166), or the interaction between the type of defoliation and the intensity of damage (*F*_3,70_ = 0.41; *p* = 0.748).

**Figure 2:**
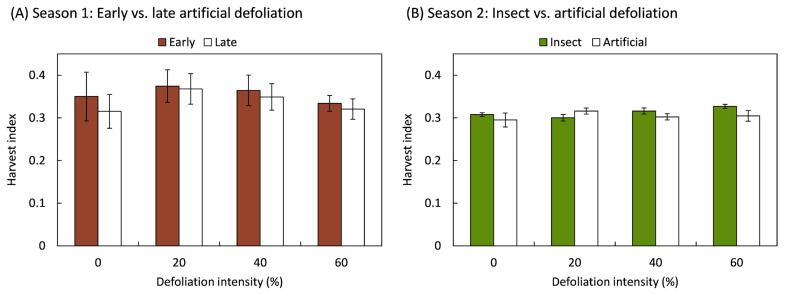
Harvest index of quinoa (*Chenopodium quinoa* Willd.) plants that were defoliated with intensities of 0, 20, 40, or 60% of leaf area removed during (**A**) early (8 BBCH-scale) or late (50 BBCH-scale) developmental stages or (**B**) subjected to artificial or insect defoliation treatments applied at the 20 BBCH-scale stage. The bars represent the average of the treatments ± 1 SEM.

Plants maintained a consistent harvest index regardless of the damage caused by the method and intensity of foliage removal (Figure 2). The harvest index showed no statistically significant variation in response to early (*F*_3,20_ = 0.19; *p* = 0.899) or late (*F*_3,20_ = 0.56; *p* = 0.647) artificial defoliations performed during the first study season, nor by the defoliation method (*F*_1,70_ = 1.64; *p* = 0.204), its intensity (*F*_3,70_ = 0.85; *p* = 0.469), or the interaction between the defoliation method and its intensity (*F*_3,70_ = 1.60; *p* = 0.197).

**Figure 3:**
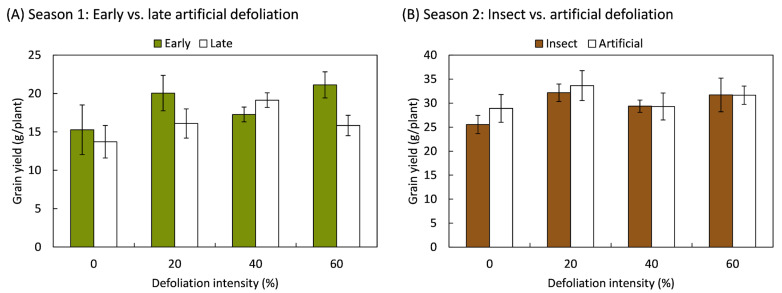
Grain yield responses of quinoa (*Chenopodium quinoa* Willd.) plants that were defoliated with intensities of 0, 20, 40, or 60% of leaf area removed during (**A**) early (8 BBCH-scale) or late (50 BBCH-scale) developmental stages or (**B**) subjected to artificial or insect defoliation treatments applied at the 20 BBCH-scale stage. The bars represent the average of the treatments ± 1 SEM.

In relation to grain yield, it was also determined that the plants compensated for the loss of foliage, such that the weight of the grains produced was not different in defoliated plants compared to those that were not defoliated. This compensatory effect was independent of the timing, intensity, and method of defoliation (Figure 3). In the first season, the timing and intensity of defoliation had no effect on the grain weight produced per plant, whether the plants had 16 leaves (8 BBCH-scale [42]) (*F*_3,20_ = 1.44; *p* = 0.260) or when the inflorescence was present, but it still was enclosed by leaves (50 BBCH-scale) (*F*_3,20_ = 1.85; *p* = 0.171). Similarly, during the second season, grain yield did not vary in response to defoliation intensity (*F*_3,70_ = 2.03; *p* = 0.118), the method of defoliation (*F*_1,70_ = 0.44; *p* = 0.510), or the interaction between both factors (*F*_3,70_ = 0.21; *p* = 0.887).

**Figure 4:**
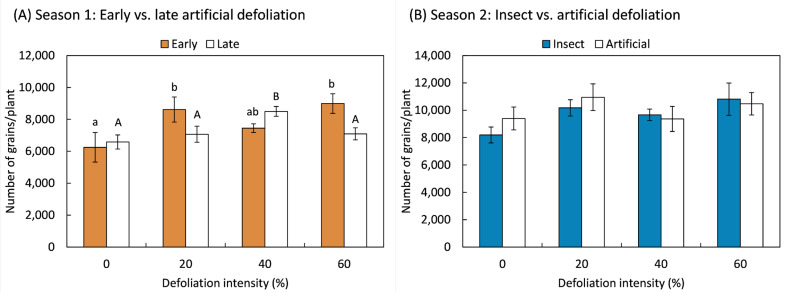
Total number of grains of quinoa (*Chenopodium quinoa* Willd.) plants that were defoliated with intensities of 0, 20, 40, or 60% of leaf area removed during (**A**) early (8 BBCH-scale) or late (50 BBCH-scale) developmental stages or (**B**) subjected to artificial or insect defoliation treatments applied at the 20 BBCH-scale stage. Different letters in the same bar (mean ± 1 SEM) indicate statistically significant differences within each defoliation moment (Fisher LSD, 0.05).

The number of grains produced further demonstrated the plants’ ability to compensate and even overcompensate for the damage caused by leaf loss. Overcompensatory responses were statistically significant in the first study season, with the number of grains increasing in response to 20% and 60% early defoliation treatments (*F*_3,20_ = 3.21; *p* = 0.045), as well as in plants defoliated later by 60% (*F*_3,20_ = 3.97; *p* = 0.023) (Figure 4A). In the second season, the number of grains produced by the plants remained unaffected by the method of defoliation (*F*_1,70_ = 0.34; *p* = 0.563), intensity (*F*_3,70_ = 2.34; *p* = 0.080), or their interaction (*F*_1,70_ = 0.45; *p* = 0.717) (Figure 4B). Although no statistically significant overcompensation effect was detected in the second season, compensatory responses were evident in both seasons, as the number of grains did not decrease as a result of the treatments.

**Figure 5:**
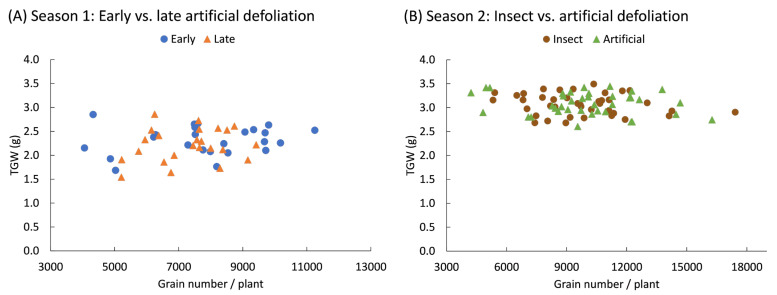
Pearson correlation coefficients between the weight of a thousand grain (TGW) and the number of grains produced by quinoa (*Chenopodium quinoa* Willd.) plants defoliated during (**A**) early (8 BBCH-scale) or late (50 BBCH-scale) developmental stages or (**B**) subjected to artificial or insect defoliation treatments applied at the 20 BBCH-scale stage. Data are presented across defoliation levels.

The weight of one thousand grains did not show a statistically significant effect from the defoliation treatments. In the first season, the weight of one thousand grains was 2.3 ± 0.05 g and did not vary based on the intensity of early (*F*_3,20_ = 0.03; *p* = 0.993) or late (*F*_3,20_ = 0.05; *p* = 0.986) defoliation. In the second season, the grain weight was 3.1 ± 0.03 g and did not vary due to the method of defoliation, whether by insects or mechanically (*F*_1,70_ = 0.08; *p* = 0.772); the intensity of defoliation (*F*_3,70_ = 1.19; *p* = 0.320); or the interaction between both factors (*F*_3,70_ = 1.02; *p* = 0.390). Additionally, the weight of the grains and the total number of grains produced by the plants did not show a statistically significant correlation (*p* > 0.05), indicating that no trade-off occurred between these two components of yield (Figure 5).

The phytochemical analysis of quinoa grains in response to the defoliation methods and intensities is summarized in Table 1. Neither the defoliation method (insect vs. artificial) nor the intensity of defoliation (0%, 20%, 40%, and 60%) significantly influenced the concentrations of the total phenols, antioxidant capacity, or total sapogenins, as indicated by the non-significant method, intensity, and interaction effects (*p* > 0.05). While total phenols ranged from 282.8 ± 11.1 to 310.0 ± 11.7 mg Tannic Acid Equivalent (TAE)/100 g dry matter (DM), no statistically significant differences were observed across treatments. Similarly, antioxidant capacity, expressed as µmol Trolox/100 g, varied between 49.9 ± 0.9 and 54.0 ± 0.8, with no significant effects of defoliation intensity (*p* = 0.304). Individual sapogenins, including hederagenin, oleanolic acid, phytolaccagenic acid, and serjanic acid, also showed no significant differences due to defoliation treatments, with hederagenin concentrations ranging from 4.6 ± 0.4 to 6.8 ± 0.8 mg/g and total sapogenins ranging from 13.1 ± 1.0 to 18.3 ± 1.8 mg/g. Although there was a trend suggesting higher oleanolic acid and phytolaccagenic acid levels in artificially defoliated plants compared to insect-defoliated plants (*p* = 0.066 and *p* = 0.077, respectively), this effect was not statistically significant. Overall, the results suggest that quinoa plants can maintain their phytochemical composition regardless of the defoliation method or intensity applied.

To verify the consistency of the application of the artificial vs. insect defoliation treatments, the leaf area of the plants was measured. Prior to the defoliation treatments, the plants had an average leaf area of 151.3 ± 2.9 cm^2^/plant. The leaf area measured 10 days after the defoliation treatments showed no significant differences in retained leaf area attributable to the defoliation method, whether artificial or natural with insects, at each defoliation level. In the control plants (no defoliation), the leaf area was 587.9 ± 67.3 vs. 559.3 ± 44.4 cm²/plant, respectively, for the artificial vs. natural defoliation with *T. ni* larvae (*T* = 0.44; *p* = 0.661). At the 20% defoliation level, the leaf area was 428.2 ± 52.7 vs. 536.3 ± 57.8 cm²/plant (*T* = −1.62; *p* = 0.111); at the 40% level, it was 387.5 ± 34.0 vs. 456.8 ± 29.1 cm²/plant (*T* = −1.06; *p* = 0.291); and at the 60% level, it was 326.4 ± 33.1 vs. 456.7 ± 39.2 cm²/plant (*T* = −1.95; *p* = 0.055), respectively, for the artificial defoliation vs. natural defoliation with *T. ni* larvae.

The plants exhibited greater growth in the second year compared to the first. Specifically, when averaging yield across treatments, it was 17.3 ± 0.7 g/plant in the first season compared to 30.2 ± 0.9 g/plant in the second season (*F*_1,124_ = 103.6; *p* < 0.001). The smaller plants from the first season experiment had a slightly higher harvest index, calculated as 0.34 ± 0.012, compared to 0.31 ± 0.003 in the second season (*F*_1,124_ = 13.27; *p* < 0.001). Similarly, the smaller plants from the first season produced fewer grains (7568 ± 232 vs. 9858 ± 293, *F*_1,124_ = 30.40; *p* < 0.001) and smaller grain size (2.3 ± 0.05 g vs. 3.1 ± 0.03 g TGW, *F*_1,124_ = 277.16; *p* < 0.001) compared to second-season plants. At the time of harvest, plant heights were 105.0 ± 1.6 cm and 111.7 ± 1.6 cm (*F*_1,124_ = 7.62; *p* = 0.007) for the first and second seasons, respectively. These differences may be attributed to the lower fertilization rate used in the first season; however, this interannual variation did not affect the conclusions of this research, as the results from both seasons consistently demonstrate that the plants tolerate defoliation.

## 3. Discussion

Investigating the level of tolerance to defoliation damage has implications for integrated pest management since the results of these investigations can be related to economic injury levels [18,43,44]. Economic injury levels refer to the amount of damage tolerated before applying a control measure [45,46]. Although knowing and applying this criterion can help reduce the use of pesticides, there is still much to be done to identify these thresholds and improve their adoption by farmers [18]. In this research, it was determined that quinoa tolerates up to 60% defoliation without affecting yield, which allows for progress in the development of a damage threshold to reduce the use of pesticides for pest control. This is relevant in quinoa production since defoliating insects are one of the main problems reported in quinoa production [5,7,8,9].

In agriculture, the relevant plant responses can be varied since the tolerance attribute is linked to the yield expected by the farmer [45]. Hence, considering that the quality of quinoa yield involves not only evaluating the total weight of the grains but also paying attention to the number and size of the grains, in situations without stress, it is common to observe a trade-off between these two factors since the plant distributes its resources selectively [47]. However, the results of this research reveal that quinoa plants managed to compensate for their performance without producing a trade-off between the size and number of grains, which is consistent with a greater phenotypic plasticity in the number of grains than in its size [29]. This result is partly explained by the increase in grain number observed, particularly in response to early foliation treatments at 20 and 60% intensity, as well as after 40% late defoliation. Coincidentally, it has been determined in other research with quinoa that the Regalona variety showed an outstanding ability to compensate for water stress by increasing the number of grains, compared to other varieties where the number of grains was reduced [48]. This aspect becomes relevant when considering that the number of grains represents one of the fundamental components of yield in quinoa [22,49]. Although Regalona was used as the model variety in this study, future research could investigate compensatory responses to defoliation in other quinoa varieties.

The effect of defoliation on plant performance may depend on the developmental stage at which it occurs. When damage occurs early, such as during the vegetative phase, plants can recover by acquiring and allocating resources to support new growth, thereby compensating for adverse effects [14,24,50]. A relevant example is amaranth *A. cruentus*, a species similar to quinoa, where it has been shown that a defoliation of up to 100% during the pre-flowering stage does not negatively affect grain yield [30,50]. In agreement, the results of the current research demonstrate that the yield in quinoa grains did not decrease due to the effect of defoliations of up to 60% in vegetative stages of 16 or 54 leaves (BBCH-scale 8 and 50, respectively [42]). In this research, defoliation stimulated the production of new leaves and stems, preventing a decrease in above-ground biomass and even leading to an increase under certain conditions, particularly during the first study season. Thus, this new growth possibly helped sustain resource acquisition, which was reflected in the unchanged yield across treatments despite defoliation.

In contrast, damage occurring during the reproductive stage could have detrimental consequences for yield, given the semi-indeterminate habit of grain formation and the fact that the greatest accumulation of biomass occurs after flowering [23,49,51]. Although specific research on quinoa in this context is limited to abiotic stresses such as drought and high temperature [48,49,52], it could be inferred that similar results of higher sensitivity to damage could manifest in response to defoliation during the reproductive stage. From an entomological perspective applied to production, it is essential to consider that many herbivorous insects feed on quinoa grains [5,7,8,9,53,54], which could imply that the plant is susceptible to the damage caused during the reproductive stage.

Among the possible mechanisms that could explain compensatory growth responses are branching, photosynthetic rate, net photosynthesis, and the mobilization and acquisition of reserves [14,19]. In another crop such as amaranth, tolerance to defoliation has been associated with the accumulation of reserves and their redistribution to produce branching and shoot regrowth [30,50]. In the specific case of quinoa, lateral branches have previously been associated with yield components, especially when the growth habit and its genotypic variability have been characterized [6,55]. Thus, it is possible that plants have modified the acquisition and translocation of resources [50], thus maintaining the ability to maintain yield in quality and quantity despite loss due to defoliation. Future research that delves into the mechanisms that allow quinoa to compensate for the adverse effects of defoliation will help understand the adaptive versatility of this plant to withstand stressful conditions.

The results of this study demonstrate that the phytochemical composition of quinoa grains remains unaffected regardless of the defoliation method (insect or artificial) or defoliation intensity (0%, 20%, 40%, or 60%). Neither the total phenolic content nor antioxidant capacity showed significant differences across treatments, suggesting that defoliation during vegetative stages does not induce these compounds. Total phenols ranged from 282.8 ± 11.1 to 310.0 ± 11.7 mg TAE/100 g DM, and antioxidant capacity varied between 49.9 ± 0.9 and 54.0 ± 0.8 µmol Trolox/100 g. Similarly, sapogenin concentrations, including hederagenin, oleanolic acid, phytolaccagenic acid, and serjanic acid, were unaffected by defoliation. Total sapogenins ranged from 13.1 ± 1.0 to 18.3 ± 1.8 mg/g, which is within the expected ranges [34,35]. Although there was a non-significant trend towards slightly higher levels of oleanolic acid and phytolaccagenic acid in artificially defoliated plants compared to insect-defoliated ones (*p* = 0.066 and *p* = 0.077, respectively), these differences were not statistically significant.

There has been growing interest in understanding plant tolerance, with evidence suggesting that low insect densities can stimulate plant growth responses, potentially benefiting food production [15,56,57]. A meta-analysis by Garcia and Eubanks [15] highlights that overcompensation is more common than previously thought, documenting 75 insect species capable of inducing such responses. For instance, potato plants (*Solanum tuberosum* L.) show overcompensatory responses when attacked at low densities by the Guatemalan potato tuber moth (*Tecia solanivora* Povolvy) [56]. In our study, overcompensation in yield was not observed; however, we detected that defoliation increased plant biomass and the number of grains under specific conditions. These findings highlight the potential for overcompensatory responses but also underscore the variability and complexity of these effects, particularly when considering their implications for overall productivity and grain quality. The context-dependence of defoliation responses and the risks associated with herbivores completing multiple life cycles further emphasize the need for careful evaluation before adopting defoliation as a stimulatory strategy.

Artificial defoliation has previously been used to simulate damage in a controlled manner across various crop species [30,43,58,59]. However, artificial damage might not fully replicate natural insect feeding, as the latter may involve specific cues or herbivore-associated elicitors that can influence plant responses, including the induction of compensatory mechanisms [56,60,61]. In our study, insect-induced defoliation achieved similar levels of intensity (20%, 40%, and 60% leaf area removed) as the artificial defoliation treatments. This allowed us to isolate the effects of defoliation intensity from those potentially attributable to the type of damage. Interestingly, we did not observe differences in plant responses based on the defoliation mechanism, suggesting that quinoa plants respond similarly to both forms of damage under our experimental conditions. However, considering the variety of herbivores that can damage quinoa, including those capable of completing multiple life cycles on the crop, further studies are needed to explore whether differences in damage type become more pronounced in response to different insect pest species.

Quinoa’s responses to herbivory remain largely unexplored compared to other plant species where saponins play a prominent defensive role. Field studies suggest that high saponin content does not protect quinoa from certain herbivores, particularly non-Lepidopteran insects [62]. Additionally, quinoa-adapted lepidoptera species have been shown to tolerate biologically relevant amounts of saponins incorporated in artificial diets [63]. In contrast, saponins in other crops, such as alfalfa (*Medicago sativa* L.), are known to deter a range of herbivores, including pea aphids [64], flea beetles [65], diamondback moths [66], armyworms [67], and European corn borers [68]. Studies on alfalfa have also shown that herbivory can induce saponin production; for example, damage by *Spodoptera littoralis* Boisduval (Lepidoptera: Noctuidae) triggered increased saponin concentrations in leaves and branches in a short-term study (<14 days) [69].

In contrast, our research, which extended over a longer period from the initial plant challenge to grain production, found no evidence of saponin induction in quinoa grains following either mechanical defoliation or damage by *T. ni* larvae. The extended interval between the initial plant challenge and grain production in our study likely contributed to this outcome, as it allowed the plants to recover fully without significantly altering grain phytochemistry. This longer period of recovery contrasts with shorter-term studies like those on alfalfa [69], where herbivory-induced saponin production occurred more rapidly. While this extended recovery period benefits grain quality, further research into the short-term effects of herbivory would provide additional insights into its potential role in enhancing insect resistance. Understanding the role of saponins in quinoa pest dynamics, particularly the difference in efficacy against specialists versus generalist pests [63], could advance pest management strategies.

## 4. Materials and Methods

### 4.1. Plants

The experiments were conducted with quinoa plants (*C. quinoa*) of the standard commercial variety Regalona Baer grown in the research facilities of the Pontificia Universidad Católica de Chile, Santiago, Chile (33°29′ S, 70°36′ W, 576 m a.s.l.). Plants were grown in individual 3 L containers with a substrate of peat (Kekkilä DSM 0, Kekkilä-BVB, Vantaa, Finland) and perlite (Harbolite^®^ A-6 Harbolite Chile Ltd.a., Santiago, Chile) in a 2:1 ratio, respectively. The plants were sown in spring and exposed to summer environmental conditions, which is their natural growth period. In the Season 1 experiment, 48 plants were sown on 1 December 2016 and harvested on 1 April 2017. The weather conditions during Season 1 included an average temperature of 21.1 °C and 53.9% relative humidity, with 28 mm of rainfall. In the experiment during Season 2, 80 plants were sown on 20 December 2017 and harvested on 6 April 2018. For this second season, the weather conditions included an average temperature of 21.0 °C and 55.7% relative humidity, with a total of 5.8 mm of rainfall. The plants were watered as needed and fertilized with nitrogen, phosphorus, and potassium (N:P:K) in the proportion of 1:2:4 plus micronutrients. In the first season, each plant was fertilized with 0.8 g N, 1.6 g phosphorus pentaoxide (P_2_O_5_), and 3.2 g potassium oxide (K_2_O) supplied with monopotassium phosphate, potassium saltpeter, and potassium sulfate. In the second season, each plant was fertilized with 1.14 g N, 2.29 g P_2_O_5_, and 4.6 g K_2_O supplied with monopotassium phosphate, potassium saltpeter, and potassium sulfate. The complete dose was divided into four parts: 10%, 35%, 35%, and 20%, each one applied at 12, 20, 30, and 40 BBCH stages [40], respectively. Microelements were supplied twice with Fetrilon (Compo Expert, Münster, Germany) (15 mg/plant) at 12 and 40 BBCH stages.

### 4.2. Defoliation Treatments

The trials of Season 1 consisted of defoliating the quinoa plants in two stages, early and late. At each time, the plants were mechanically defoliated at different levels: 0%, 20%, 40%, and 60%. For this, the plants were separated into two groups of 24 each, one group being used for early defoliation and the other for late defoliation. Defoliation was carried out at two phenological moments of the plant, both before panicle emission. The first defoliation (early defoliation) was carried out when the plants had an average of 16 true leaves (BBCH-scale 8) [42]. The second defoliation (late defoliation) was performed when the inflorescence was present, but it was still enclosed by leaves (BBCH-scale 50) [42]; at this stage, plants had an average of 54 leaves. The plants were defoliated mechanically with scissors to achieve targeted defoliation levels, which were estimated by counting the total number of leaves and then removing whole leaves according to the desired defoliation percentage.

Experiments conducted during the second season involved three levels of defoliation: 20%, 40%, and 60%, plus a control group with no defoliation (0%). For each level of insect defoliation, either cabbage looper larvae (*T. ni*) or artificial defoliation (using scissors) was employed. The defoliation treatments were applied when the plants reached the stage of 22 expanded leaves, corresponding to growth stage 20 [42].

For insect-induced defoliation, *T. ni* larvae were obtained from a colony reared on quinoa plants under greenhouse conditions. Each plant was infested with second and third instar larvae, with 6, 11, and 22 larvae used per plant to achieve defoliation levels of 20%, 40%, and 60%, respectively. The larvae were kept on the plants for four days, and larvae were removed and replaced as necessary to visually achieve the desired defoliation percentages. The plants were covered with chiffon mesh to keep the insects contained. All plants, including the control and mechanically defoliated plants, were kept under mesh for the same duration as those defoliated with insects. Mechanical defoliation treatments involved removing leaves from the plants with scissors, as performed during the first season experiments.

At the end of the defoliation treatments, the meshes covering the plants were removed, and all plants were sprayed with insecticide and fungicide. The insecticide lambda-cyhalothrin (Karate Zeon 050 CS, Syngenta, Santiago, Chile) was applied at a concentration of 0.5 mL formulated product (f.p.)/L, and the fungicides Mefenoxam and Mancozeb (Ridomil Gold MZ 68 WG, Syngenta, Santiago, Chile) were applied at a concentration of 2.5 g f.p./L to eliminate all larvae and prevent mildew. On two occasions, the fungicides Ridomil and Iprodione (Iprodion 50 WP, Agrospec, Santiago, Chile) were applied to control quinoa downy mildew and Alternaria.

The leaf area was measured ten days after the treatments began during the second study season by counting the total number of leaves on each plant using templates that matched the different leaf sizes found in quinoa plants. The area of the template leaves was analyzed using WCIF Image J v1.37 software (Wright Cell Imaging Facility, University Health Network Research, Toronto, ON, Canada). The leaf area of plants defoliated by insects was measured using a modified scale designed for assessing the severity of downy mildew fungus on tomato leaflets [70], which closely resembles the larval feeding patterns observed on quinoa leaves. Additionally, the height of the plants was measured at the time of harvest.

### 4.3. Plant Responses to Defoliation

Plants of both experimental seasons were harvested when the stem color turned from yellow to brown and all leaves were dead (BBCH-scale 95 [42]. For each plant, the entire aerial part (leaves, stems and grains) was dried in an oven at 60 °C for 72 h. The grains were then separated from the leaves and stems, and their weight was recorded for each plant individually. Grain yield was determined as the total dry weight of grains produced by each plant (g/plant). The weight of a thousand grains (TGW) was determined for each plant [71]. The number of grains per plant was determined by dividing yield weight by average grain weight. The harvest index was calculated as the total dry weight of grains produced by each plant divided by the total dry weight of the aerial part (leaves, stems, and grains).

Grains harvested from the second season were subjected to analyses of total phenols, antioxidant capacity, and the content of total and individual sapogenins, including oleanolic acid (OA), hederagenin (HE), phytolacagenic acid (PA), and serjanic acid (SA). The grains were ground using a micro-plant grinding machine to pass through a 1 mm screen and were stored in a desiccator until analysis.

To analyze the concentration of total phenols and the antioxidant capacity of the quinoa grains, a subsample of 40 mg of ground grains was weighed for each treatment, and a double extraction was carried out with 0.5 mL of methanol for 24 h each (1 mL total solvent). The total phenol content was measured using the Folin–Ciocalteu reagent, following the method described by Tang et al. [72], with some modifications [73,74,75], in a PowerWave HT 96-well plate reader (BioTek Instruments, Winooski, VT, USA). The Folin–Ciocalteu reagent (diluted 1:10 with distilled water) and 7% *w*/*v* sodium carbonate (Na_2_CO_3_) were added to the diluted extract of quinoa grains. The mixture was left in the dark at room temperature for 90 min. Tannic acid was used as the standard phenolic compound. The absorbance was then measured using the plate reader at a wavelength of 725 nm. The results were expressed as milligrams of tannic acid (TA) per gram of dry weight of quinoa grain extract (mg TA/100 g DW grain extract) using the calibration curve obtained with tannic acid (r² = 0.99).

The antioxidant capacity of quinoa grains was determined using the DPPH reagent method, with some modifications [32,72], in a PowerWave HT 96-well plate reader (BioTek Instruments, Winooski, VT, USA). The DPPH reagent was added to the diluted extract of the quinoa grains at a concentration of 0.06 mM. The mixture was left in the dark at room temperature for 30 min. Trolox was used as the standard compound. Absorbance was measured at a wavelength of 517 nm. The antioxidant capacity of each extract was expressed as µmol of Trolox equivalent (TE) per gram of extract dry weight (µmol TE/100 g DW) using the calibration curve determined with Trolox (r² = 0.998).

The sapogenin content was determined by high-performance liquid chromatography (HPLC, Dionex UltiMate 3000, Thermo Fisher Scientific, Waltham, MA, USA) according to previously described methods [34,63]. An extraction with hexane was performed on 75 mg of grain powder, followed by three methanol extractions. The extracts were resuspended in water, and a hydrolysis reaction was performed using hydrochloric acid (HCl). The hydrolysis product was partitioned with chloroform, and the organic phase was washed with water and dried. The residue was redissolved in methanol and stored at −20 °C. Before chromatography, the samples were centrifuged, transferred to HPLC vials, and dried. They were then redissolved in a mixture of acetonitrile and water using a water bath and sonicator and immediately analyzed by HPLC. Commercial and isolated standards were used to quantify the sapogenins. Total sapogenins and four individual sapogenins—hederagenin, oleanolic acid, phytolacagenic acid, and serjanic acid—were measured. The results were expressed as milligrams of sapogenin per gram of dry weight of grain extract (mg/g DW grain extract).

### 4.4. Data Analysis

In the first season experiment, two groups of plants grown at the same time were used, with one group that was defoliated at an early stage of development and another at a more advanced stage. The experimental design of each group was completely randomized, with six replicates for each treatment. Statistical analysis of the results of each group was performed independently using a one-way analysis of variance. The experiment conducted in the second season was designed as a factorial experiment with defoliation type (artificial or with insect larvae) and defoliation intensity considered as fixed main factors (*n* = 10). Data were analyzed using a generalized linear model (GLM). Differences between treatments were analyzed using a least significant difference test (Fisher LSD, 0.05). The relationships between dependent variables, TGW, and grain number, were quantified with Pearson correlation coefficients.

## 5. Conclusions

This research demonstrated that quinoa can tolerate various levels and timings of defoliation without negatively impacting its yield in both quantity and quality. Depending on the intensity and timing, grain number even increased, showing overcompensatory responses. The method of defoliation, either mechanical or using *T. ni* larvae, did not influence the overall plant responses in yield. These compensatory responses highlight the resilience of quinoa under stress conditions, which aids in sustainable food production.

## Figures and Tables

**Table 1:** Phytochemistry of quinoa (*Chenopodium quinoa* Willd.) grains in relation to defoliation method and intensity. Total phenols (mg TAE/100 g DM), antioxidant capacity (µmoles Trolox/100 g), hederagenin (HE) (mg/g), oleanolic acid (OA) (mg/g), phytolaccagenic acid (PA) (mg/g), serjanic acid (SA) (mg/g), and total sapogenins (mg/g). Values are means ± 1 SEM.

Defoliation		Total Phenols	Antioxidant Capacity	HE	OA	PA	SA	Total Sapogenins
Method	Intensity (%)							
Insect	0	282.8 ± 11.1	53.2 ± 1.9	5.1 ± 0.4	5.4 ± 0.3	3.2 ± 0.2	0.7 ± 0.03	14.3 ± 0.9
	20	291.8 ± 15.8	49.9 ± 0.9	4.8 ± 0.6	4.9 ± 0.5	3.0 ± 0.3	0.6 ± 0.05	13.3 ± 1.5
	40	292.8 ± 15.1	53.8 ± 1.2	5.9 ± 1.0	5.6 ± 0.5	3.2 ± 0.3	0.7 ± 0.05	15.5 ± 1.6
	60	310.0 ± 11.7	54.0 ± 0.8	4.6 ± 0.4	4.8 ± 0.3	3.0 ± 0.3	0.6 ± 0.04	13.1 ± 1.0
Artificial	0	297.6 ± 15.4	52.1 ± 1.3	5.1 ± 0.6	5.5 ± 0.5	3.1 ± 0.3	0.7 ± 0.06	14.4 ± 1.4
	20	300.3 ± 7.4	51.3 ± 1.2	5.6 ± 0.6	5.8 ± 0.5	3.5 ± 0.4	0.7 ± 0.05	15.5 ± 1.5
	40	301.9 ± 14.8	50.2 ± 0.8	6.8 ± 0.8	6.7 ± 0.6	4.0 ± 0.4	0.8 ± 0.04	18.3 ± 1.8
	60	283.5 ± 18.1	51.4 ± 1.4	5.1 ± 0.4	5.2 ± 0.4	3.3 ± 0.3	0.7 ± 0.05	14.3 ± 1.1
Method effects		*F*_1,70_ = 0.02 *p* = 0.881	*F*_1,70_ = 2.87 *p* = 0.095	*F*_1,70_ = 1.70 *p* = 0.197	*F*_1,70_ = 3.48 *p* = 0.066	*F*_1,70_ = 3.21 *p* = 0.077	*F*_1,70_ = 1.47 *p* = 0.230	*F*_1,70_ = 2.73 *p* = 0.103
Intensity effects		*F*_3,70_ = 0.11 *p* = 0.953	*F*_3,70_ = 1.23 *p* = 0.304	*F*_3,70_ = 2.41 *p* = 0.074	*F*_3,70_ = 2.26 *p* = 0.089	*F*_3,70_ = 0.97 *p* = 0.410	*F*_3,70_ = 1.11 *p* = 0.353	*F*_3,70_ = 2.08 *p* = 0.110
Interaction effects		*F*_3,70_ = 0.88 *p* = 0.455	*F*_3,70_ = 1.61 *p* = 0.195	*F*_3,70_ = 0.18 *p* = 0.910	*F*_3,70_ = 0.50 *p* = 0.686	*F*_3,70_ = 0.58 *p* = 0.631	*F*_3,70_ = 0.31 *p* = 0.817	*F*_3,70_ = 0.37 *p* = 0.773

## Data Availability

The data presented in this study are available on request from the corresponding author.
